# Hypertension awareness, treatment and control in Africa: a systematic review

**DOI:** 10.1186/1471-2261-13-54

**Published:** 2013-08-02

**Authors:** James Kayima, Rhoda K Wanyenze, Achilles Katamba, Elli Leontsini, Fred Nuwaha

**Affiliations:** 1 Department of Medicine, School of Medicine, College of Health Sciences, Makerere University, P.O. Box 7072, Kampala, Uganda; 2 Department of Disease Control and Environmental Health, School of Public Health, College of Health Sciences, Makerere University, P.O. Box 7072, Kampala, Uganda; 3 Clinical Epidemiology Unit, Department of Medicine, School of Medicine, College of Health Sciences, Makerere University, P.O. Box 7072, Kampala, Uganda; 4 Department of International Health, Johns Hopkins Bloomberg School of Public Health, Baltimore, MD 21205, USA

**Keywords:** Hypertension, Awareness, Control, Gender, Associated factors

## Abstract

**Background:**

Inadequate diagnosis and suboptimal control of hypertension is a major driver of cardiovascular morbidity and mortality in Africa. Understanding the levels of awareness, treatment and control of hypertension and the associated factors has important implications for hypertension control efforts.

**Methods:**

The PubMed database was searched for original articles related to awareness, treatment and control of hypertension in Africa published between 1993 and 2013. The key search terms were: Africa, awareness, treatment, control, and hypertension. Exploration of bibliographies cited in the identified articles was done to provide further studies. Full texts of the articles were obtained from various internet sources and individual authors. A data extraction sheet was used to collect this information.

**Results:**

Thirty eight studies drawn from 23 African countries from all regions of the continent met the inclusion criteria. The levels of awareness, treatment and control varied widely from country to country. Rural populations had lower levels of awareness than urban areas. North African countries had the highest levels of treatment in the continent. There was generally poor control of hypertension across the region even among subjects that were aware of their status and those that were treated. On the whole, the women had a better control status than the men.

**Conclusion:**

There are low levels of awareness and treatment of hypertension and even lower levels of control. Tailored research is required to uncover specific reasons behind these low levels of awareness and treatment, and especially control, in order to inform policy formulation for the improvement of outcomes of hypertensive patients in Africa.

## Background

Cardiovascular disease (CVD) is the leading cause of death in developing countries where it causes nearly as many deaths as HIV, malaria and tuberculosis [1,2]. Africa, a continent of 53 countries and one billion people is not only beleaguered by a huge burden of infectious disease but also bears a considerable proportion of the world’s CVD burden [3]. Presently, the age-specific mortality rates from CVDs are much higher in younger age groups in both men and women in Africa than in the developed world [4]. CVD is the second leading overall cause of death in Africa, after HIV/AIDS, and is the leading cause of mortality among individuals over the age of thirty [5]. The World Health Organisation (WHO) projects that over the next ten years Africa will experience the largest increase in death rates from CVD and therefore the negative economic impact of CVD will be more felt on the continent [3].

Hypertension is the driver of the CVD epidemic in Africa where it is a major, independent risk factor for heart failure, and stroke and kidney failure [6]. The management of these complications is difficult to sustain in sub-Saharan countries where resource-intensive care is not very feasible. Insufficient diagnosis of hypertension and suboptimal blood pressure control in the diagnosed patients increases morbidity and mortality with an increased burden to health care resources [7]. In developed countries, the improved control of hypertension has led to considerable reduction in overall morbidity and mortality over the last fifty years [8,9]. Evidence from large clinical trials has shown a 40% reduction in stroke and a reduction of at least 25% in myocardial infarction associated with treatment and control of hypertension [10,11].

The increasing awareness of hypertension as an important cause of morbidity and mortality in Africa over the last twenty years has resulted in numerous publications documenting the burden of hypertension in Africa which now calls for systematic reviews to provide a comprehensive understanding of the situation in order to inform the design of tailored control and research efforts. In 2007, Addo et al. published a systematic review which showed extensively varying prevalence of hypertension with higher prevalence rates in the urban than rural areas in Sub-Saharan. There was marginal reporting on the levels of awareness, treatment or control in that publication [12]. Since then, there have been several studies conducted that have further highlighted the challenge of awareness, treatment and control of hypertension. This systematic review is therefore intended to evaluate the available data on levels of awareness, treatment and control of hypertension in Africa with a view of suggesting measures that could improve control among hypertensive patients.

## Methods

This systematic review followed the guidelines set out by Preferred Reporting Items for Systematic reviews and Meta-Analyses (PRISMA) statement [13]. We considered articles published in all languages between 1993 and 2013 to give a more comprehensive and current appraisal of awareness, treatment and control of hypertension in Africa. Journal articles in other languages were translated to English. To ensure relative homogeneity and comparability of the studies, we considered only community based cross-sectional studies that reported awareness, treatment and control of hypertension. We considered articles that defined hypertension as a measured blood pressure of >140 systolic and >90 diastolic; awareness as the prior knowledge of hypertensive status; treatment as any attempted pharmacological therapy for hypertension in patients who were aware of their status and control as a blood pressure of <140 systolic and <90 mmHg diastolic in treated hypertensive subjects. We included studies that used both automated and mercury sphygmomanometers as blood pressure measuring devices, and surveys that had a sample size of at least 350 participants. Non-pharmacological therapy is an important way of controlling hypertension and indeed a first line for pre-hypertension. However, the studies included, described only control with pharmacological therapy because this is a more demonstrable way of therapy. We excluded studies in which hypertension status was determined using one blood pressure reading.

We searched in PubMed using the key search terms: Africa, awareness, treatment, control, and hypertension. We also used individual country names for the 53 African countries and hypertension as additional key search terms to obtain articles on the subject. Eligible articles were assessed by one member of the team (JK). We excluded all studies that were hospital based and all data whose definitions of awareness, treatment and control differed from the agreed-on definitions. The abstracts of all potentially relevant papers were reviewed and full articles were accessed through Pub Med or Google Scholar or HINARI. The references of all the relevant research articles were scrutinised for additional potential data sources. The full texts of these studies were also accessed in a similar way. The authors whose full texts papers could not be accessed by the numerous internet based sources used were requested to provide them.

A data extraction sheet was used to collect information about the country, the year of publication, the number of participants, the prevalence of hypertension and the levels of awareness, treatment or control. Where the factors that predicted the levels of awareness, treatment and control were provided, these too were extracted. Where possible, results from multinational studies were disaggregated to show the status of awareness, treatment and control within individual countries. Where it was not possible to disaggregate the data by country, the study was presented as one and the countries in which the study was done were shown. In the publications considered, level of awareness was measured as a proportion of the total numbers of hypertensive subjects that were aware of their hypertensive status; level of treatment as a proportion of the subjects who were aware of their hypertensive status who were on pharmacological therapy and level of control as the proportion of treated patients who attained a blood pressure of 140/90 mmHg. The threshold statistical significance for factors that predicted hypertension was a p-value < 0.05. We also recorded odds ratios in those publications that reported them. Owing to the heterogeneity of the study designs and the lack of reported confidence intervals in most the studies, a combined analysis of the reported data could not be done.

## Results

**Figure 1:**
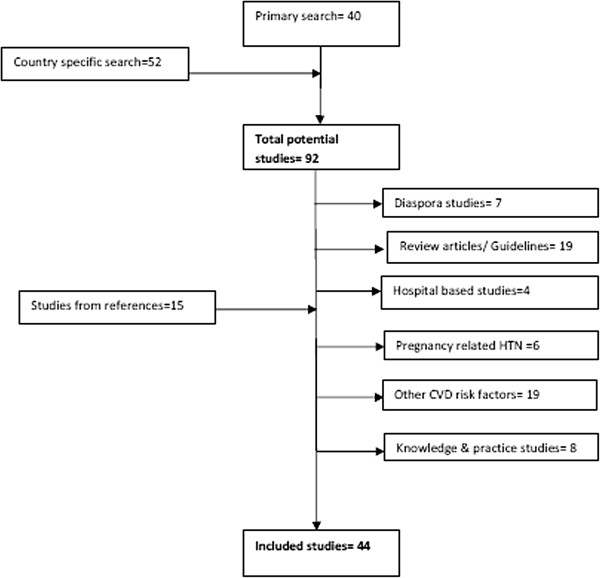
Flow chart of literature search.

Figure 1 shows the schema of the process of choosing the studies that were included in the review. The primary PubMed search identified 40 articles. The country-specific searches led to the identification of an additional 52 studies. We excluded six studies that considered maternal hypertension; eight that described awareness as “knowledge of the effects of hypertension” rather than “awareness of hypertensive status”; 19 review papers and guidelines and 6 studies among Africans in the diaspora (living outside the continent). Fifteen additional studies were extracted from the bibliographies of the eligible articles and included for review. Eventually 44 studies were included in the review of the awareness, treatment and control of hypertension in Africa.

**Figure 2:**
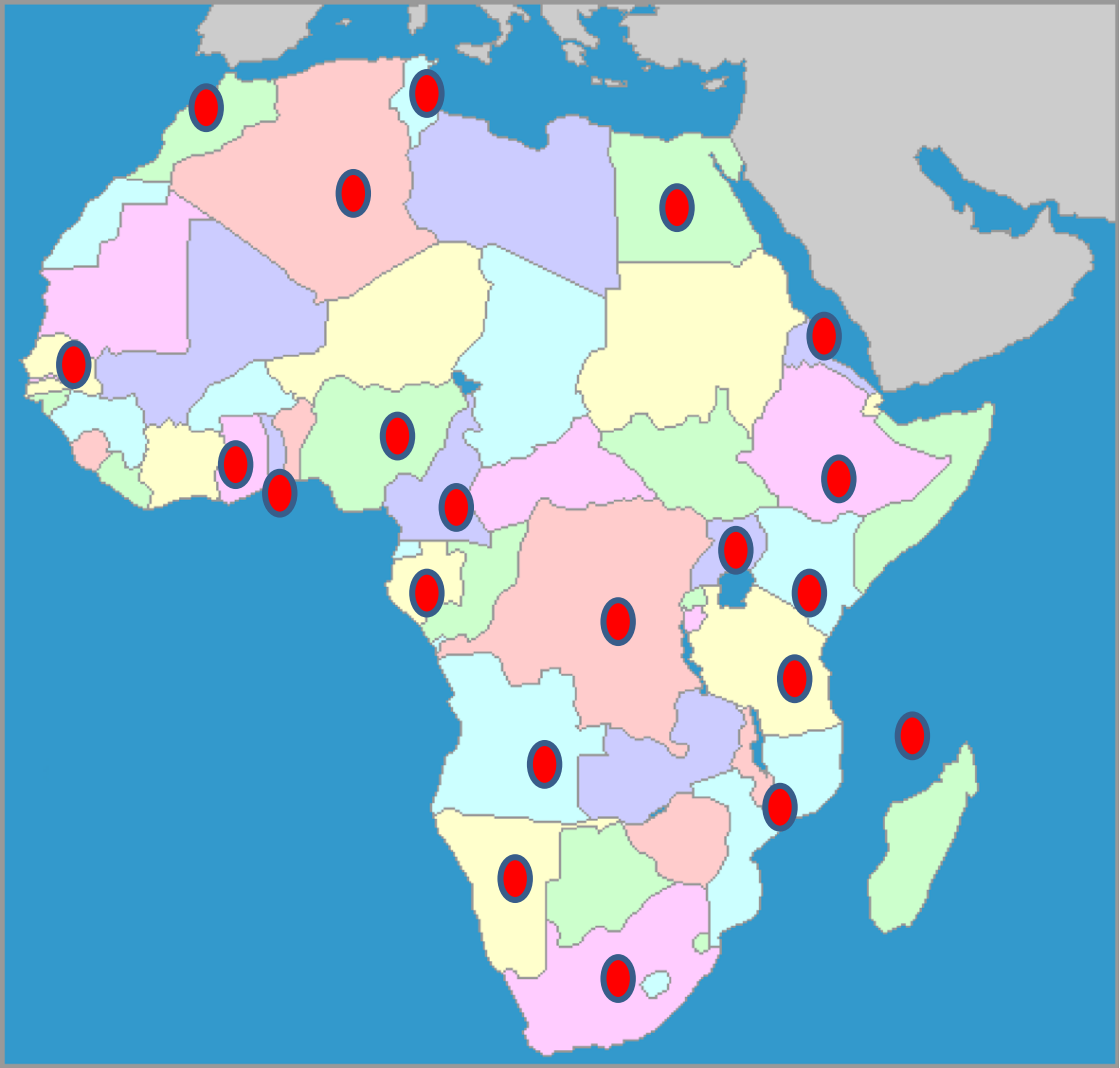
Map of Africa distribution of studies reviewed.

Figure 2 shows the countries on the African continent represented in the review. Overall, 26 of the 53 African countries were represented from all the regions of the continent. East Africa was represented by fourteen studies from seven countries [14-27]. Central Africa [28-33] and North Africa [34-39] were each represented by six studies. West Africa was represented by eleven studies [21,40-49]. An additional four studies from South Africa and Namibia were included in the survey [21,50-52].

Table 1 summarises the findings of the systematic review including any factors associated with diagnosis, treatment and control where available. The populations represented in this review were diverse and included both rural and urban subjects. Two of the articles were from multinational studies [21,35]. An additional six studies were nation-wide survey [29,30,36,37,53,54]. The special communities represented in the studies were slum dwellers in Kenya, gold miners in South Africa and the market community in Nigeria [19,44,50].

**Table 1:** Results of systematic review of hypertension awareness, treatment, control, and any associated factors from 44 studies in Africa, 1993-2013

**Country**	**Location**	**Age range**	**Sample size**			**Prevalence (%)**				**Factors associated with treatment, awareness and control**
			**Males**	**Female**	**Total**		**Aware (% of hypertensive)**	**Treated (% of aware)**	**Control (% treated)**	
**Comoros**										
Solet JL , 2008 [14]	Islands	30–69			1268	44.0	33.0		27.0	Elderly more aware than the young
**Eritrea**										
Mufunda, 2006 [15]	Rural and Urban	15–64	1160	1182	2342	15.6	20.0			
**Ethiopia**										
Muluneh AT, 2012 [16]	Rural and urban	15–64	2084	2385	4469	9.3			27.0	
Awoke A , 2012 [17]	Urban	>35			679	28.3	63.0		42.2	Obesity and low physical activity associated with poor control
Tesfaye F, 2009 [18]	Urban	25–64	1398	1875	3713	31.5	35.2	11	28.0	
**Kenya**										
van de Vijver SJ, 2013 [19]	Urban slums	>18	2794	2396	5190	12.3	19.5	47	21.0	
Jenson A, 2011 [20]	Coastal area		162	307	469	43.1	46.5	31.2	7.3	Women more aware OR 4.58 (1.81–11.64); better treated OR 4.02(1.89–8.54) and controlled OR 8.1(1.81–11.64) Older >60 more aware OR6.01(1.11–32.48), better treated OR3.22(1.00–10.32) and controlled 3.22(1.04–9.99)
Hendriks EM, 2011 [21]	Rural	>18	847	1264	2111	21.4	17.0	9	8.0	
**Mozambique**										
Damasceno et al., 2009 [22]	Rural and urban	25–64	1281	1800	3081	33.1	14.8	48.1	39.9	Women more aware (*p <* 0.007), better treated *(P <* 0.021) and better controlled (*p <* 0.001)
Seychelles										
Bovet et al., 2004 [23]	Nationalwide survey		1255	568	1823	39.2	65.0	62	24.1	Women more aware, treated and controlled
**Tanzania**										
Dewhurst et al., 2012 [25]	Elderly	>70	972	1251	2223	70.0	37.7	6.1	0.9	Men better treated, women more controlled
Hendriks EM, 2011 [21]	Urban	>18	423	623	1046	23.7	18.0	9	5.0	
Bovet P.,2002 [24]			9254	3659	5645	29.1	31.0	11	6.5	Women, better educated well controlled
Edwards R et al., 2000 [26]	Rural and urban	>15	402	526	928	30.0	20.0	10	<1.0	No difference in rural and urban
**Uganda**										
Musinguzi, 2013 [27]	Rural	>15	4563	2940	1623	27.2	28.2	51.6	9.4	Women more aware, controlled
**Angola**										
Pires JE, 2013[28]	Mainly urban	18-64	853	611	1,464	23.0	21.6	13.9	33.0	Awareness, higher in women (p < 0.001); older subjects (p < 0.0001); non-smokers (p < 0.009). Lower in heavy alcohol drinkers (p < 0.007)
**Cameroon**										
Kamadjeu RM et al., 2006 [29]	Nationwide survey	>15	4007	6004	10,011	24.1	23.0	40.0		Older age, longer duration of HTN predicted awareness and treatment
Dzudie A et al., 2012 [30]	Nationwide screen	>15	2120	1117	1,003	47.5	37.1	59.9	24.6	
**DRC. (Kinshasa)**										
Katchunga PB, 2011 [31]	Rural and urban	>20			699	40.2	42.5	30.5	13.6	
**Gabon**										
Ngoungou EB, 2012 [32]		>40	313	423	736	51.9	9.9	19.4	5.8	
**Zimbambwe**										
Matenga, 1996 [33]		> 34			749	33.3	26.1	22.4	47.8	
**Algeria**										
Temmar M, 2007 [34]	Urban > rural	40–99	637	711	1348	44.0		30.0	25.0	Women controlled better than men (p < 0.001)
Nejjari C et al., 2013 [35]	Multinational	>18	27296	17339	10292	45.4	7.0	91.2	37.5	Control better in urban areas (p < 0.00001), younger (p < 0.01), female(p < 0.01); university level (p < 0.00001); non-smokers (p < 0.0001); those with normal BMI (p < 0.00001); normal cholesterol level (p < 0.01)
**Egypt**										
Ibrahim MM, 1995 [36]	Nationwide survey	25–95	2928	3804	6733	36.3	37.5	23.9	8.0	Better awareness, treatment and control in urban vs. rural. Better awareness and treatment in those with severe HTN. Better awareness and control in women and elderly
**Tunisia**										
Romdhane BH, 2012 [37]	Nationwide survey	35–70			8007	30.6	38.8	84.4	24.1	Awareness better in older age group, females, lower education, urban subjects
Hammami, 2011 [38]	Urban elderly	>65	202	396	598	52.0	81.0	78.0	30.7	Awareness better in elderly population, those with free medications & the insured
Romdhane BH, 2005 [38]		40–60			1837	44.0	41.0	74.0	18.0	
**South Africa**										
Maepe LM, 2012 [50]	Gold miners	18–69	3867	430	4297	39.5		42.0	31.0	
Malhotra R, 2008 [51]			107	530	637	40.0	49.2			Younger age associated with better awareness; 35–54 yrs [OR 0.33 (0.19–0.57)] and 55 yrs [OR 0.27 (0.14–0.54)] c.f age 18–34
Steyn K, 2008 [52]	Nationwide		5738	8064	13801	21.0	M-41 F-67	M-39 f-58	M-26 F 38	Better control predicted by SES: richest vs poorest OR 2.33(1.17–4.62) ; Race asian vs black OR 1.76(1.03–3.02); Age: oldest age group vs youngest OR 26.23 (4.92–141); Insurance: non-insured vs insured OR 0.59 (0.38–0.91)
**Namibia**										
Hendriks EM, 2011 [21]	Urban	>18	771	962	1733	38.0	38.0	17	12	
**Benin**										
Houinato DS, 2012 [40]			3461	3392	6853	27.9	22.5	4.8	1.9	
**Ghana**										
Agyemang, 2006 [41]	Rural urban	>16	787	644	1431	29.4	34.0	28	6.2	Awareness better in older age (≥50-yr olds cf 16-34-yr olds [OR = 6.14, (2.98–12.64). Treatment better in older age ≥50-year-olds OR: 6.25 ( 2.87–13.62); overweight OR 1.85(1.02, 3.34); traders OR 2.51(1.03–7.40]) Control better in traders OR 2.51 (1.03–7.40) but poor in old age: ≥50-year-olds OR 0.11( 0.01–0.60)
Addo, 2006 [42]	Rural	>18	107	255	362	22.4	33.2	16.7		
Amoah, 2003 [63]	Urban	>25	1893	2840	4733	28.3	34.0	18	4.0	
**Nigeria**										
Ulasi II, 2011 [44]	Market community		379	352	731	54.2	49.8			
Oladapo OO, 2010 [45]			873	1127	2000	39.2	14.2	18.6		Transport, lack of drugs at health units, competing priorities
Hendriks EM, 2011 [21]	Rural, multinational	>18	1247	1431	2678	19.3	8.0	5.0	3.0	
Omuemu, 2007 [46]	Rural				590	20.2	18.5	77.3	29.2	Women more aware, educated, elderly
Ekwunife, 2010 [47]		>18	364	392	756	21.1	30.0	21.0	9.0	Men more aware, women more controlled
**Senegal**										
Macia E, 2012 [48]	Urban	>50	263	237	500	64.5	49.5	70.6	17.4	Better awareness for women OR 2.4(1.41–4.07); elderly > 70 yrs OR 2.15(1.11–4.17) but worse for the educated > 8 years OR 2.15 (1.01–4.6 ); those who had not visited HCW within the year OR 0.37 ( 0.23–0.6) Better treatment and control for those who had visited HCW within the last year (P < 0.001)
**Togo**										
Yayehd K, 2011 [49]			2002	1095	905	36.7	42.4			

*HTN* hypertension, *HCW* Health care workers, *SES* socioeconomic status, *BMI* body mass Index, *OR* (95% CI)- Odds ratio followed by 95% CI.

The total number of participants in the review was 121, 220. The sample size ranged from 375 subjects in a market place in Nigeria [44] to 27296 in a multinational study in North Africa [35]. The age of the participants ranged from 15 years to 99 years. Five studies focused on the middle aged and elderly populations above forty years [25,32,34,38,39]. All the studies had both genders (male and female) represented. In most of the studies, the women participants were more than the men. In studies that targeted special communities- gold miners, slum dwellers and market communities- the men had more representation.

### Prevalence of hypertension

There was a wide variation in prevalence of hypertension. Most of the prevalence rates were not age-standardised which made comparisons difficult. Among the studies that considered younger populations under 35 years, the prevalence ranged from 9.3% in an Ethiopian population to 48.1% in a Mozambican population [16,22]. As expected, those studies that involved the elderly populations had higher prevalence of hypertension reaching 70% in an urban Tanzanian population aged more than 70 years [25].

### Awareness

Awareness in this review was described as prior knowledge of hypertensive status. The lowest levels of awareness were found in rural communities in Nigeria (8%), Uganda (10%) and Gabon (9%)[21,27,32]. The lowest prevalence of awareness in urban areas was 12.3% among slum dwellers in Nairobi. The highest awareness rates were found in the studies that considered elderly subjects reaching 81% in urban elderly populations of Tunisia [39]. Generally, studies from North African countries showed the highest levels of awareness. The large multinational Epidemiological Trial of Hypertension in North Africa (ETHNA) that included 27296 subjects revealed awareness rate of 71% among hypertensive patients [35]. West and central Africa seemed to have the lowest levels of awareness of hypertension status.

### Treatment

The treatment of hypertension ranged from 5% in a rural Nigerian community to 91.2% in urban North African populations [21,35]. East African populations had the lowest levels of treatment while North African countries had the highest levels. In studies done in Tanzania, for example, the treatment rates ranged between 6.1% and 11% whereas those done in North Africa varied between 24% in an Egyptian national wide survey and 91.2% in the ETHNA study [24,25,35,36]. There was no clear difference in treatment rates between urban and rural populations across the regions.

### Control rates

Despite varying rates of awareness and treatment, the control rates were uniformly low and never exceeded 45%. Tanzanian populations- both urban and rural- had the lowest levels ranging from as low as <1% and never exceeding 6.5% in all the four studies that spanned twelve years. As with awareness and treatment rates, the North African studies showed the highest rate of control. The least levels of control in North Africa were recorded in Egypt at 8% with the highest recorded in Morocco, Tunisia and Algeria in the multinational ETHNA study [35,36].

### Factors affecting treatment awareness and control

Only 24 out of the 44 studies attempted to review any factors associated with awareness, treatment and control status. In all the studies, the older age group had better awareness and treatment rates. However, this did not universally translate into better control of the blood pressure. In fact, two studies reported better control in the young hypertensive patients [35,41]. Additionally, most studies revealed better prevalence of awareness, treatment and control among the women. Even the two studies that showed better awareness of hypertensive status in men eventually revealed superior treatment and control status in the women [25,47].

All the studies that had a comparison of rural and urban dwellings showed generally better hypertension treatment and control status in the urban areas. Additionally, subjects with better formal education and literacy levels seemed to have better control. While socio-economic status was not a frequently recorded variable in the studies, the patients who had health insurance or who had access to private care had better control [52]. Moreover North African countries, most of which have access to universal health insurance had better detection and treatment profiles, although these did not translate into better control rate [39].

## Discussion

There has been a wealth of information on the prevalence of hypertension on the African continent. A 2007 systematic review by Addo et al. had illustrated the high prevalence of hypertension in numerous African states [12]. Although our search terms did not include prevalence, this systematic review reveals varied levels of prevalence across countries, a great many of which confirm persisting high rate. In a high prevalence setting like Africa, it is of utmost importance to describe not only the detection rate but also awareness, treatment and control as well as the factors that influence these rates. This would enable the formulation of relevant tailor-made control strategies in order to reduce the complications of uncontrolled hypertension. This systematic review is, to our knowledge, the most comprehensive analysis on awareness, treatment and control on the African continent to date.

Our results show generally low levels of awareness of hypertensive status with North African countries having relatively better levels. The levels in Africa are much lower than those in North America and Europe where temporal reviews have shown an improvement in awareness from twenty years ago when the levels where similar to the level currently seen in Africa to the present rate of over 65% [53]. Most of this improved awareness has been attributed to rigorous education programs on hypertension after the realization that hypertension was a major player in morbidity and mortality in these countries. Whereas the heterogeneous nature of the designs of the study reviewed here could not allow the establishment of a temporal trend in this review, no improvement was realised in countries that had multiple consecutive studies such as Tanzania. It is possible that similar appreciation of hypertension as leading cause of death would lead to improved education and therefore improved awareness.

The treatment rates found by our review in North Africa were much higher than those found elsewhere on the continent. It is possible that the availability of health insurance in countries such as Tunisia which covers both diagnosis and treatment steers these high levels of treatment. In Massachusetts USA, universal health insurance coverage has led to improved diagnosis and control of hypertension leading to a reduction in hypertension related hospitalisations and deaths [54]. It is possible that hypertension status will improve in countries aiming to attain universal health coverage such as South Africa. In other parts of Africa, nationalized health insurance remains out of reach. Most Africans pay out-of-pocket for their health expenses, which are supplemented somewhat by a few free services from government and donor organizations which mainly focus on infectious disease treatment with HIV/AIDS control efforts taking a lion’s share of this funding [55]. This calls for more innovative ways of financing care for chronic non-communicable disease on the continent.

The studies considered in this review revealed generally dismal rates of control of hypertension. The lowest rates of control were seen in Tanzania in studies spanning 10 years. Even in countries that had impressive rates of treatment, control of blood pressure to target remained elusive in many. This means that treatment of hypertension does not guarantee control, the ultimate predictor of outcomes. Several of the included studies from different countries blamed various factors for poor blood pressure control. These can generally be categorised as such as the health-system deficiencies, patients’ non-adherence and the physicians’ inertia in treating hypertension and they seem to be in play on the African continent. For example, the lack of anti-hypertensive medication at health facilities and the long distance to the health facilities reported in some studies are characteristic health system shortcomings hindering the achievement of control [28,44]. On the other hand the lack of time and reported competing priorities are classical patient and physician factors adding to the problem [45].

Blood pressure in women was generally better controlled than in men in Africa. In two studies in Tanzania and Nigeria, the men were more aware of their status and were more likely to receive treatment. Still, the women achieved better control rates. The issue of gender differences in hypertension has been a subject of intense research. It is now recognised that young men are more likely to develop hypertension than women, a dynamic that has been blamed on androgen mediated abnormalities in pressure natrieusis [56]. However, large meta-analyses of hypertension treatment trials have failed to document gender differences in response to antihypertensive medication meaning that the poor control of hypertension documented in men is probably due to socio-economic and cultural factors [57]. In other parts of the world, control of blood pressure seems to be poorer in women. This is possibly because these studies have largely considered elderly men and women [58,59]. In our review women of all age groups seemed to have better control. In various studies in the African setting, the women seem to have better health seeking behaviour for chronic disease than men [60,61]. It is also possible that women benefit from screening due to more contact with healthcare facilities during the reproductive years [62]. The men, on the other hand, are less enthusiastic in seeking care. Indeed in one included study from Angola, men reportedly did not pick up their antihypertensive medication because they lacked time [28]. The finding supports the establishment of gender-specific programs for the treatment and control of blood pressure.

There were certain limitations in this review. The use of one database may have limited the number of articles obtained for the review. However, by searching the PubMed database we limited our search to peer-reviewed sources which offered a firm guarantee that the publications used were appropriate for review [63].

As previously mentioned, these studies were heterogeneous in nature which made further analysis difficult. In addition, the cross-sectional nature of the considered studies complicated efforts to discuss the trends in awareness, treatment and control. In Tanzania, frequent studies have been done in the capital Dar es Salaam over time and so we commented on trends [24,26]. There is need to carry out surveillance or follow-up cohorts in order to study the trends of hypertension status. A further limitation with most of the reviewed studies was the failure to study the factors that contributed to the current hypertension status on the continent which calls for systematic study of these factors to inform interventions and policy. Other draw-backs included non-random selection of participants in some of the studies [30,44] the under-representation of the central African region.

## Conclusion

This systematic review confirms the low levels of treatment and awareness. Most notably, the level of control is abysmal in most countries in Africa, which implies that diagnosis and treatment does not guarantee reaching targets of control. This situation explains the poor outcomes of patients affected with hypertension on the continent. While health system factors play a key part in perpetuating this state of affairs, patient factors are also important determinants of control. These factors may differ in gender and on different parts of the continent necessitating tailored research and policy formulation to improve the outcomes of patients affected by hypertension. In addition, there is need to design tailored chronic care models on a continent whose focus has thus far been the control of acute infectious disease.

## Competing interests

The authors declare that they have no competing interests.

## Authors’ contribution

JK designed the review, conducted the literature search and drafted the first version of the manuscript. All authors read and approved the manuscript and contributed to its content.

## Pre-publication history

The pre-publication history for this paper can be accessed here:

http://www.biomedcentral.com/1471-2261/13/54/prepub
